# Whole Genome Sequencing and Comparative Genomic Analysis of *Pseudomonas aeruginosa* SF416, a Potential Broad-Spectrum Biocontrol Agent Against *Xanthomonas oryzae* pv. *oryzae*

**DOI:** 10.3390/microorganisms12112263

**Published:** 2024-11-08

**Authors:** Yikun Zhang, Zhongfeng Zhu, Tian Qin, Xiaojuan Li, RuoChen Yu, Zifan Tang, Chenjiayi Zhang, Yichao Yan, Ke Yin, Zhengyin Xu, Gongyou Chen, Lifang Zou, Youlun Xiao

**Affiliations:** 1 Shanghai Collaborative Innovation Center of Agri-Seeds, School of Agriculture and Biology, Shanghai Jiao Tong University, Shanghai 200240, China; zyk982833375@sjtu.edu.cn (Y.Z.); zhuzhongfeng@sjtu.edu.cn (Z.Z.); renjianshigeer@sjtu.edu.cn (T.Q.); qq345559686@sjtu.edu.cn (Z.T.); zhangchenjiayi2004@sjtu.edu.cn (C.Z.); yichao.yan@sjtu.edu.cn (Y.Y.); yin.ke@sjtu.edu.cn (K.Y.); xuzy2015@sjtu.edu.cn (Z.X.); gyouchen@sjtu.edu.cn (G.C.); 2 Institute of Plant Protection, Hunan Academy of Agricultural Sciences, Changsha 410125, China; 3 School of Agriculture, Ningxia University, Yinchuan 750021, China; 4 State Key Laboratory of Microbial Metabolism, Shanghai Jiao Tong University, Shanghai 200240, China

**Keywords:** *Xanthomonas oryzae* pv. *oryzae*, *Pseudomonas aeruginosa*, biological control agents, whole genome sequencing, comparative genomic analysis

## Abstract

Rice is one of the most important staple crops worldwide. However, the bacterial blight of rice caused by *Xanthomonas oryzae* pv. *oryzae* (*Xoo*) poses a major threat to the production of rice. In this study, we isolated and identified the strain *Pseudomonas aeruginosa* SF416, which exhibited significant antagonistic activity against *Xoo*, from a soil sample collected in a winter wheat field in Shannanzhalang County, Tibet, China. The bacterial solution (BS) and cell-free supernatant (CFS) of SF416 had significant prevention effects for the bacterial blight of rice, with an efficacy of 45.1% and 34.18%, respectively, while they exhibited a slightly lower therapeutic efficiency of 31.64% and 25.09%. The genomic analysis showed that *P. aeruginosa* SF416 contains genes involved in cell motility, colonization, cold and hot shock proteins, antibiotic resistance, and plant growth promotion. SF416 also harbors two sets of phenazine-1-carboxylic acid (PCA) synthesis gene clusters, phz1 (*phzA1*-*G1*) and phz2 (*phzA2*-*G2*), and other phenozine product-synthesis--related genes *phzS*, *phzM*, and *phzH*, as well as genes in the SF416 genome that share high similarity with the ones in the genomes of *P*. *aeruginosa* M18, suggesting that the two sets of PCA synthesis gene clusters are responsible for the antagonistic effect of SF416 against *Xoo*. A comparative antiSMASH analysis revealed that *P*. *aeruginosa* SF416 contains 17 gene clusters related to secondary metabolite synthesis, 7 of which, encoding for pyochelin, azetidomonamide A/B, L-2-amino-4-methoxy-trans-3-butenoic acid, hydrogen cyanide, pyocyanine, pseudopaline, and bicyclomycin, are conserved in strains of *P*. *aeruginosa.* Moreover, SF416 can produce protease and siderophores and display a broad-spectrum antagonistic activity against various major plant pathogenic bacteria and fungi. The results suggest that *P*. *aeruginosa* SF416 could be a potential candidate agent for the bacterial blight of rice.

## 1. Introduction

Rice is one of the most important staple crops globally, accounting for about one-third of the world’s primary food source. Its cultivation and production are crucial for the food security and livelihoods of billions of people [1]. However, rice production is often threatened by various diseases, with bacterial diseases being particularly severe. Bacterial blight of rice caused by *Xanthomonas oryzae* pv. *oryzae* (*Xoo*) is one of the most devastating and widespread diseases affecting rice [2]. This disease severely affects the rice yield and quality, thereby posing a major threat to agricultural economies [3]. It is estimated that bacterial blight of rice causes approximately 20% yield loss annually in Asian rice fields [4]. Thus, controlling bacterial blight of rice is essential for maintaining stable global rice production and ensuring food security [5].

Currently, the control of bacterial blight of rice in production practice mainly relies on the use of resistant varieties and chemical bactericides. The discovery of resistance genes during the breeding process of disease-resistant varieties is fundamental to achieving success [6]. To date, researchers have identified 47 resistance genes against bacterial blight of rice in both cultivated and wild rice, with 31 being dominant and 16 recessive, and 17 of these genes have been cloned [7,8]. In 2020, Chen et al. identified the Xa46(t) gene, which showed resistance to all pathogenic types found in China [9]. *Xa23*, isolated from wild rice (*Oryza rufipogon*), exhibits high resistance to nearly all natural *Xoo* strains tested, likely due to the prevalence of its corresponding *avrXa23* gene in these natural strains [10]. However, regional variation in bacterial pathogens and the fact that some high-yielding and superior rice varieties lack these resistance genes present challenges for disease control. Therefore, chemical control is needed in some areas where bacterial blight of rice occurs heavily. Chemical control mainly depends on bactericides such as copper agents, zinc agents, and seboctylamine, but their long-term use can lead to environmental pollution and the development of pathogen resistance [11]. For example, while copper agents are effective at inhibiting pathogens, they pose significant pollution risks to soil and water. Consequently, environmentally friendly biological control strategies have gradually become a focus of research.

In recent years, the application of biocontrol bacteria in managing rice diseases has gained increasing attention. Numerous studies have demonstrated the promising potential of using microorganisms and their metabolic products to control rice diseases. Research has shown that various *Streptomyces* spp. and *Bacillus* spp. exhibit significant inhibitory effects on *Xanthomonas oryzae*, the pathogen responsible for rice diseases [12,13]. For instance, *Bacillus velezensis* FZB42 has been shown to secrete multiple antibacterial substances that effectively inhibit the growth of *Xoo* [14]. *Bacillus amyloliquefaciens* Lx-11 can secrete 3 lipopeptide compounds, which are effective in suppressing rice streak disease. Therefore, Lx-11 is considered to be a promising biocontrol agent [15]. Similarly, *Bacillus velezensis* HN-2 produces surfactin, a compound with strong antibacterial activity against *Xoo*, which not only inhibits its growth, but also induces rice resistance to *Xoo* by mediating antioxidant-related enzymes [16].

*Pseudomonas* spp. has also shown broad application prospects in biocontrol. For example, *Pseudomonas mosselii* 923 produces the antagonistic compound pseudoiodinin, which exhibits strong inhibitory effects against *Xoo*, *Xanthomonas oryzae* pv. *oryzicola* (*Xoc*), and *M. oryzae* [17]. Additionally, *Pseudomonas* fluorescens has been proven to inhibit *Xoo* through mechanisms such as inducing plant disease resistance [18]. Overall, biocontrol strategies can significantly reduce the use of chemical agents, protect the environment, and delay the development of pathogen resistance, making them an essential direction for future rice disease management [19]. Integrating agricultural, chemical, and biological control methods to comprehensively manage rice diseases will enhance the sustainability and safety of rice production [20].

In this study, we report on a strain of *Pseudomonas aeruginosa* SF416, which was isolated and screened from soil in a winter wheat field in Shannan Zhalang County, Tibet, China. *P. aeruginosa* SF416 exhibited significant in vitro inhibitory effects against *Xoo*, as well as broad-spectrum antagonistic activity against various major plant pathogenic bacteria and fungi. Field experiments further showed that *P. aeruginosa* SF416 had a therapeutic effect on bacterial blight of rice. Additionally, we analyzed the whole genome sequence of *P. aeruginosa* SF416, focusing on genes involved in plant growth promotion, environmental adaptability, and secondary metabolite gene clusters. Overall, our findings suggest that *P*. *aeruginosa* SF416 could be a potential candidate agent for the bacterial blight of rice.

## 2. Materials and Methods

### 2.1. Strain Screening, Preservation, and Determination of Minimum Inhibitory Concentration (MIC)

In this study, we screened 27 candidate bacterial strains previously preserved in the laboratory using the wild-type strain of *Xoc* RS105 as the target bacterium of antagonism. We used the plate confrontation method to identify potential biocontrol agents. *Xoc* RS105 and the candidate strains were inoculated into Nutrient Broth (NB) medium under conditions of 28 °C and 180 rpm for 12–24 h. The OD_600_ was then adjusted to 2.0. Subsequently, 200 µL of the *Xoc* RS105 bacterial suspension was added to melted and appropriately cooled Nutrient Agar (NA) solid medium and thoroughly mixed. Ensure that the temperature of the medium is 45–50 °C when mixing. Once solidified, place an Oxford cup (8 mm diameter) in the center of the plate. Candidate bacterial strains was inoculated into NB medium and cultured overnight at 28 °C, 200 rpm. The OD_600_ was then adjusted to 1.0. An amount of 50 µL of the candidate strain suspension was added to the Oxford cup, and the plates were incubated under 28 °C for 48 h. The inhibition zones were measured and recorded. Inhibition rate = (diameter of inhibition circle—diameter of Oxford cup)/diameter of inhibition circle × 100%. The strain with the highest inhibition rate was selected, as the study strain and plate standoff experiments were completed against a variety of plant pathogenic bacteria including *Xoo* PXO99^A^, *Xoc* RS105, *X. citri* subsp. *citri*, *X. campestris* pv. *musacearum, X. campestris* pv. *malvacearum*, *X. axonopodis* pv. *glycines*, *X. campestris* pv. *vesicatoria*, *X. campestris* pv. *phaseoli*, *X. axonopodis* pv. *vignicola*, *X. axonopodis* pv. *vasculorum*, *X. axonopodis* pv. *allii*, *X. campestris* pv. *juglandis*, *Acidovorax citrulli*, and *Pseudomonas syringae* pv. *tomato* DC3000. The selected strain was then cultured in NB medium under conditions of 28 °C and 180 rpm for 12 h. Afterward, 1 mL of the bacterial culture was mixed with 1 mL of sterile 50% glycerol and stored at −80 °C for long-term preservation.

Determination of minimum inhibitory concentration (MIC): Incubated pathogenic bacteria in NB medium at 28 °C with 200 rpm for 24 h. The concentration of the bacterial solution was then adjusted to 10^5^ CFU/mL. We incubated SF416 in NB medium at 28 °C with shaking at 200 rpm for 48 h. The culture was then centrifuged at 10,000 rpm for 5 min to remove the precipitate. The supernatant was concentrated using a freeze dryer, reducing the volume to one-tenth of the original. Before use, the supernatant was filtered through a 0.22 μm bacterial filter. In a 96-well plate, 50 μL of the pathogen culture and 50 μL of the concentrated SF416 supernatant were added to each well. The SF416 supernatant was diluted in a two-fold gradient, and the final MIC (minimum inhibitory concentration) was determined based on the concentration of the supernatant in the solution system.

Formulation for media used in the article: Nutrient Broth (NB) medium: 5 g/L polypeptone, 10 g/L sucrose, 1 g/L yeast extract, 3 g/L beef extract; Nutrient Agar (NA) medium: add 15 g/L agar to the NB medium formulation; Potato Dextrose Agar (PDA) medium: 6 g/L potato extract, 20 g/L dextrose, 20 g/L agar.

### 2.2. 16S rRNA and Whole Genome Sequencing

The genomic DNA of strain SF416 was extracted using the Omega Bacteria DNA Kit. The *16S rRNA* gene was amplified using PCR using the universal primers 27F (5′-AGAGTTTGATCCTGGCTCAG-3′) and 1492R (5′-TACGGCTACCTTGTTACGACTT-3′) [21]. The PCR products were sent to Biosune Biotechnology (No.69, Lane 1985, Chunshen Road, Minhang District, Shanghai, China ) for sequencing analysis.

The strain was inoculated into NB medium and cultured overnight at 28 °C, 200 rpm. The culture was then diluted 1:100 into fresh NB medium and grown until OD_600_ of the cell density reached 0.6. The cells were harvested using centrifugation at 6000 r/min at 4 °C for 10 min and then the supernatant was discarded. The pellet was washed with 1× PBS buffer, and this process was repeated until the supernatant was clear. After removing all the supernatant, the pellet was frozen in liquid nitrogen for 15 min and stored at −80 °C. The samples were then sent to Personalbio (Building 2, No. 218 Yindu Road, Xuhui District, Shanghai, China) for whole genome sequencing using the Illumina NovaSeq and PacBio Sequel platforms.

### 2.3. Phylogenetic Tree Analyses

The *16S rRNA* gene sequence was subjected to BLAST analysis on NCBI to determine the species of closely related bacteria. Using MEGA11 software, closely related strains were selected for comparison, and the *16S rRNA* sequence of SF416 was used to construct a neighbor-joining phylogenetic tree. Based on the phylogenetic tree constructed from the *16S rRNA* sequences, closely related strains were selected, and their whole genome data were downloaded from NCBI. The assembled whole genome sequencing results of SF416 and the selected closely related strains were used to construct a whole genome phylogenetic tree through the TYGS online platform (https://tygs.dsmz.de/, accessed on 7 March 2024.) [22]. ANIb and DDH analyses were performed using the online platforms (http://jspecies.ribohost.com/jspeciesws/, accessed on 28 May 2024.) and (https://ggdc.dsmz.de/, accessed on 20 April 2024), respectively [23,24].

### 2.4. Plate Inhibition Experiment and Biological Control Experiment in Paddy Field

The determination of the inhibition rate of plant pathogenic bacteria is similar to the screening of biocontrol strains, as described in Section 2.1. The determination of inhibition rate of plant pathogenic fungi is different. We punched holes in the medium where the fungus was growing with an 8 mm hole punch to obtain a round fungal cake. The fungal cake was placed in the center of the PDA medium, and filter paper was placed 2 cm from the center of the plate. An amount of 5 µL of SF416 bacterial solution was added dropwise to each filter paper sheet. The control group was titrated with NB medium. Each group of 3 replicates was cultured at 28 °C for 5–7 d. Finally, the fungal growth diameters were recorded, and the corresponding inhibition efficiencies were calculated. Inhibition efficiency = (control fungal growth diameter − experimental fungal growth diameter)/control fungal growth diameter × 100%. Plant pathogenic fungi used in the experiment include *Magnaporthe oryzae*, *Fusarium oxysporium*, *Botrytis cinerea*, *Fusarium graminearum*, *Phytophthora capsica*, and *Colletotrichum gloeosporioides*. Plant pathogenic bacteria include *X*. *citri* subsp. *citri*, *X*. *campestris* pv. *musacearum*, *X*. *campestris* pv. *malvacearum*, *X*. *axonopodis* pv. *glycines*, *X*. *campestris* pv. *vesicatoria*, *X*. *campestris* pv. *phaseoli*, *X*. *axonopodis* pv. *vignicola*, *X*. *axonopodis* pv. *vasculorum*, *X*. *axonopodis* pv. *allii*, *X*. *campestris* pv. *juglandis*, *Acidovorax citrulli*, and *Pseudomonas syringae* pv. *tomato* DC3000.

Biocontrol assays in paddy fields were performed as in our previous study [17]. Xoo was inoculated in NB medium and incubated overnight at 28 °C with 200 rpm. The optical density at 600 nm (OD_600_) was adjusted to 0.3 before being sprayed on the leaves of the susceptible rice variety, IR24, at the seedling stage. Ten replicates were performed for each treatment. We developed the preventive (Pre) and therapeutic (Tre) treatments in which the rice leaves of IR24 were inoculated with the bacterial solution (BS) and cell-free supernatant (CFS) of *Pseudomonas aeruginosa* SF416 at 24 h before inoculation with *Xoo* PXO99^A^ (Pre) or 24 h after inoculation with *Xoo* PXO99^A^ (Tre). At the same time, BS and CFS were replaced by zinc thiazole as positive control, and water was replaced by BS and CFS as negative control. We incubated SF416 in NB medium at 28 degrees with 200 rpm for 24 h, adjusting OD_600_ = 1.0 to obtain the BS of SF416; it was incubated for 48 h, centrifuged at 10,000 rpm for 5 min to remove the precipitate, and passed through a 0.22 μ bacterial filter to obtain the CFS of SF416. The disease indexes of bacterial blight of rice using the different treatments were investigated 15 days after inoculation.

### 2.5. Protease and Siderophores Detection Assay

Extracellular protease capacity assay medium: 0.1 g/mL skimmed milk powder solution, 0.03 g/mL agar solution, 0.1 mmol/L phosphate buffer (pH = 7), respectively, were autoclaved at 121 °C for 20 min and mixed thoroughly according to the ratio of 10:10:3 when used.

Chromium azurite CAS medium: 20 mL of CAS A solution, 2 mL of CAS B solution, 0.8 g of sucrose, 1.2 g of acid-hydrolyzed casein, 8 mL of 1 mmol/L MgSO_4_ solution, 0.4 mL of 1 mmol/L CaCl_2_ solution, and 8 g of agar were dissolved with deionized water; finally, volume was fixed to 400 mL and autoclaved at 121 °C for 20 min (CAS A solution: 1 mmol/L CAS, 4 mmol/L HDTMA, 0.1 mmol/L FeCl_3_; CAS B liquid: 0.1 mmol/L phosphate buffer, pH 7.0).

Using the Oxford cup method, SF416 was cultured in NB medium overnight, adjusted OD_600_ = 2.0, inoculated on CAS and extracellular protease assay medium, respectively, and then observed and photographed after 48 h of culture.

### 2.6. Genome-Wide Assembly and Annotation

The company Shanghai Paisano Biotechnology completed the second and third generation molecular sequencing. The third-generation single molecule sequencing data were assembled by using HGAP (v4) CANU (v1.7.1) to obtain contig sequences from the offline data obtained using Pacbio. The raw offline data (raw data) from second-generation molecular sequencing was filtered to generate high-quality sequences (high-quality data). Subsequently, the second-generation high-quality data were corrected against the third-generation contig results using pilon software (v1.18) and finally spliced to obtain the complete sequence [25].

GeneMarkS software (v4.32) was used to predict protein-coding genes across the entire genome [26]. tRNA genes were predicted using tRNAscan-SE [27], and rRNA genes were identified with Barrnap. The remaining non-coding RNAs were predicted by comparison with the Rfam database [28]. PhiSpy was used to predict prophages present in the genome [29], IslandViewer 4 to identify genomic islands [30], and CRISPR finder to locate DRs (direct repeats) and spacers in the genome [31]. The assembled sequences were functionally annotated using several databases: GO (Gene Ontology), KEGG (Kyoto Encyclopedia of Genes and Genomes), COG (Clusters of Orthologous Groups), Swiss-Prot, NR (Non-Redundant Protein Database), CAZy (Carbohydrate-Active enZYmes Database), CARD (The Comprehensive Antibiotic Resistance Database), and antiSMASH [32,33,34]. These annotations were carried out using the Swiss-Prot database. The annotation of Swiss-Prot for protein-coding genes was performed using diamond software (v0.8.36). We used diamond blastp to compare the sequences of the encoded proteins with those in the database, and the critical value of sequence comparison was chosen as 1 × 10^−6^; the functional discrimination rule of the sequences was: E-value < 1 × 10^−6^, and the Swiss-Prot name with the best hits was assigned to the corresponding protein-coding genes.

Availability of data and materials: The complete genome sequence has been uploaded to the NCBI database with the following information: BioProject: PRJNA970529; BioSample: SAMN35005130; Genomes: CP125288.2.7. 

Comparative genomics analysis: We selected four closely related strains, *P. aeruginosa* PAO1, *P. aeruginosa* M18, *P. aeruginosa* DN1, and *P. otitidis* MrB4, for comparative genomic analysis with SF416. Genome-wide comparisons were performed and genome-wide comparative circle maps were plotted through BRIG 0.95 and BLAST+ software (v2.15.0) [35]. Covariance analyses of the whole genome data were performed using Mauve software (v2.4.0), and visualization images were drawn [36]. The whole genome sequence files were uploaded to the antiSMASH online website for secondary metabolite prediction, and gene cluster comparison images were drawn using PowerPoint. The orthologous clusters comparison was performed through OrthoVenn3 (https://orthovenn3.bioinfotoolkits.net/ accessed on 28 April 2024) [37].

### 2.7. Statistical Analysis

Significant differences between means were compared by using the LSD test (least significant difference test) at *p* = 0.05. *p* value < 0.05 was considered significant.

## 3. Results

### 3.1. Screening and Identification of Strain SF416 That Exhibits Highly Antagonistic Activity Against Xanthomonas oryzae pv. oryzae

**Figure 1:**
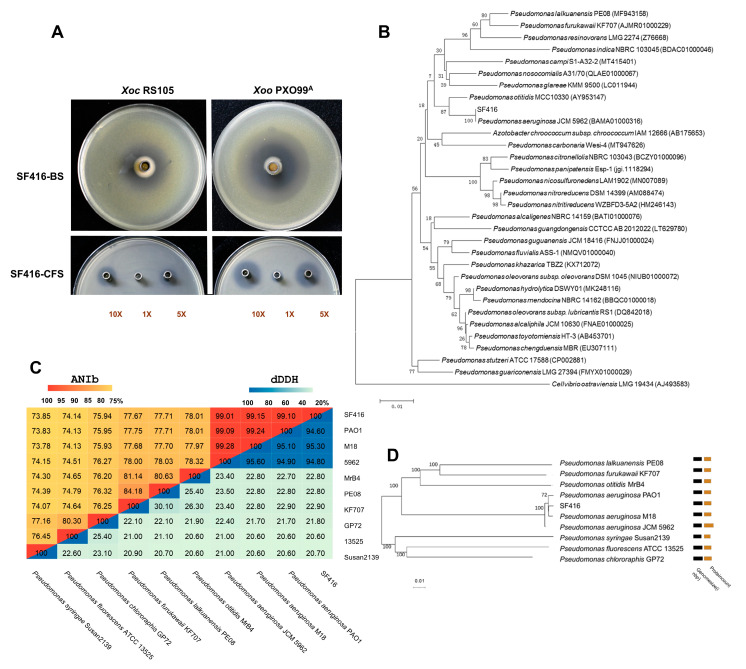
Screening and identification of strain SF416, which exhibits antagonistic activity against *Xoo* and *Xoc*. (**A**) Inhibitory effect of the bacterial solution (BS) and the cell-free supernatant (CFS) of SF416 against *Xoo* PXO99^A^ and *Xoc* RS105. (**B**) Phylogenetic evolutionary tree for SF416 based on the *16S rRNA* sequences. (**C**) The ANIb and DDH values of SF416 with nine *Pseudomonas* strains, including three *Pseudomonas aeruginosa* strains. (**D**) Phylogenetic evolutionary tree of the whole genome for SF416.

In a previous study, we attained 223 bacterial isolates that exhibited antagonistic activity against the *Xoc* wild-type strain RS105 from the 248 rhizosphere soil samples [38]. In this study, we tested the antibacterial activity of additional 27 candidate strains that were previously preserved in our laboratory and screened from 58 of the 248 rhizosphere soil samples. We found that one strain, SF416, showed significant antagonistic activity against *Xoc* RS105 and *Xoo* PXO99^A^ with inhibition ratios of 84.74% and 79.61%, respectively (Figure 1A); however, the antibacterial activity against *Xoo* PXO99^A^ by SF416 was significantly stronger than *Xoc* RS105 (Table 1). The cell-free supernatant (CFS) of SF416 also exhibited antagonistic activity against *Xoo* PXO99^A^ and *Xoc* RS105, and the 10-fold concentrated CFS showed 76.16% and 62.48% inhibitory rates against *Xoo* PXO99^A^ and *Xoc* RS105, respectively (Figure 1A and Table S1). The minimum inhibitory concentration (MIC) of SF416 CFS against *Xoo* PXO99^A^ and *Xoc* RS105 were also determined to be 309.1 µg/mL (0.25×) and 618.2 µg/mL (0.5×), individually. After purification, SF416 forms faint yellow and slightly raised colonies when cultured on an NA medium, and microscopic observation revealed rod-shaped cells (Figure S1). Strain SF416 has been preserved in the China Center for Type Culture Collection (CCTCC) under the accession number M2023164. Additionally, SF416 exhibited broad-spectrum antimicrobial properties, inhibiting ten plant pathogenic *Xanthomonas* species, including *X*. *citri* subsp. *citri* (*Xcc*), two important plant pathogenic bacteria, including *Acidovorax citrulli* causing bacterial fruit blotch and *Pseudomonas syringae* pv. *tomato* DC3000 (Table 1 and Figure S2), as well as six plant pathogenic fungi, including *Magnaporthe oryzae*, *Fusarium oxysporium*, *Botrytis cinerea*, *Fusarium graminearum*, *Phytophthora capsici*, and *Colletotrichum gloeosporioides* (Table 1 and Figure S3).

To determine the taxonomic position of SF416, we constructed a phylogenetic tree using the *16S rRNA* sequence. The results showed that SF416 clustered in the same branch as *Pseudomonas aeruginosa* JCM 5962 (Figure 1B). We further selected nine *Pseudomonas* strains and performed ANIb and dDDH analyses on their whole genome sequences, constructing a phylogenetic tree. The results indicate that SF416 had ANIb and dDDH values above the commonly accepted species threshold (ANI ≥ 95%, DDH ≥ 70%) with three *Pseudomonas aeruginosa* strains, and the ANIb and dDDH values between SF416 and the other six *Pseudomonas* strains were below the species threshold (Figure 1C). Among them, SF416 was most closely related to *Pseudomonas aeruginosa* M18, with ANIb and dDDH values of 99.15% and 95.30%, respectively (Figure 1C,D). M18 has proved to be an effective biocontrol agent with broad-spectrum antimicrobial activity against *Xoo*, the Gram-positive bacterium *Bacillus cereus*, and some plant pathogenic fungi [39,40]. These results further confirm that SF416 belongs to *Pseudomonas aeruginosa*.

### 3.2. Assessment of SF416 as Effective Biocontrol Agent for Bacterial Blight of Rice

**Figure 2:**
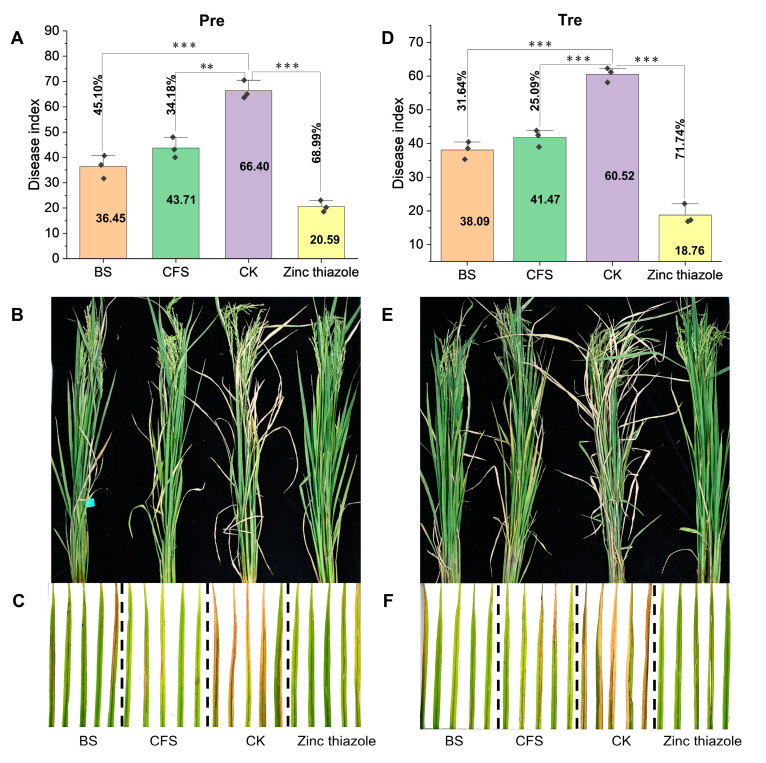
The preventive and therapeutic effects of the BS and CFS of *P. aeruginosa* SF416 against bacterial blight of rice. (**A**) The application of SF416 BS and CFS resulted in a significant reduction in the disease indexes in the Pre treatment. (**B**) The bacterial blight incidence of a representative rice plant in the Pre treatment. (**C**) Phenotypes of representative rice leaves infected with *Xoo* PXO99^A^ in the Pre treatment. (**D**) The application of SF416 BS and CFS resulted in a significant reduction in the disease indexes in the Tre treatment. (**E**) The bacterial blight incidence of a representative rice plant in the Tre treatment. (**F**) Phenotypes of representative rice leaves infected with *Xoo* PXO99^A^ in the Tre treatment. ** *p*-value < 0.01; *** *p*-value < 0.001.

To evaluate the potential of SF416 for the control of bacterial blight of rice, we performed a spray inoculation experiment on leaves of the susceptible rice variety IR24 in a paddy field. The wild-type *Xoo* PXO99^A^ without the BS and CFS of *P. aeruginosa* SF416 treatments (the control group, CK) could successfully infect the leaves of IR24, causing disease indexes of 66.40 and 60.52 in the Tre and Pre treatments, respectively (Figure 2A,D). In the treatment of the chemical agent zinc thiazole, disease indexes of 20.59 and 18.76 were acquired, indicating a prevention efficiency of 68.99%, and a therapeutic efficiency of 71.74%, respectively (Figure 2A,D). Compared with CK, the leaf blight areas of individual leaves infected with *Xoo* PXO99^A^ were significantly reduced in the Pre treatment, and only disease indexes of 36.45 and 43.71 were acquired using the BS and CFS of *P. aeruginosa* SF416 treatments, indicating a prevention efficiency of 45.1% and 34.18%, respectively (Figure 2A–C). In the Tre treatment, disease indexes of 39.09 and 41.47 were acquired using the BS and CFS of *P. aeruginosa* SF416 treatments, indicating a therapeutic efficiency of 31.64% and 25.09%, respectively (Figure 2D–F), indicating that the effect of the Pre treatment is slightly better than that of the Tre treatment. These results showed that *P. aeruginosa* SF416 has potential as a biocontrol agent for managing the bacterial blight of rice; however, the biological control effect needs to be further improved.

### 3.3. Genomic Features and Functional Gene Analysis of P. aeruginosa SF416

**Figure 3:**
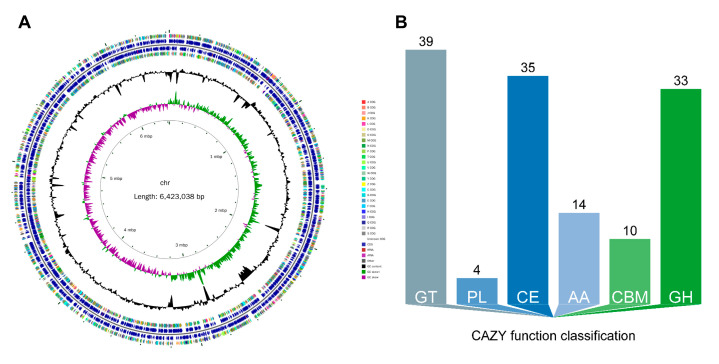
General genome features of *P. aeruginosa* SF416. (**A**) SF416 genome circle diagram: from inside out, the first circle represents the scale, the second circle represents the GC Skew, the third circle represents the GC content, the fourth and seventh circles represent the COGs to which each CDS belongs, and the fifth and sixth circles represent the positions of CDS, tRNA, and rRNA on the genome. (**B**) The carbohydrate-active enzymes (CAZymes) in the SF416 genome predicted using the Carbohydrate-Active enZYmes Database (CAZy).

The genome of SF416 consists of a single circular chromosome with a total length of 6,423,038 bp (Figure 3A) and a GC content of 66.23%. The predicted number of coding sequences (CDS) in SF416 is 5785, which accounts for 87.97% of the chromosome length. Among these, 632 CDS contain signal peptide sequences, 522 are predicted to encode secreted proteins, and 1318 CDS contain at least one transmembrane helix region. Additionally, the genome of SF416 includes 65 tRNA copies, 12 rRNA copies, 76 snRNA copies, 9 prophages, 33 genomic islands, and 6 CRISPR structures (Table 2). Functional annotation of the CDS in SF416 was performed using various databases, resulting in the annotation of 5739 (99.20%) genes in the NR database, 5219 (90.22%) in the eggNOG database, 3297 (56.99%) in the KEGG database, 4345 (75.11%) in the Swiss-Prot database, and 4351 (75.21%) in the GO database.

The Comprehensive Antibiotic Resistance Database (CARD) was used to predict antibiotic resistance genes in SF416. A total of 78 genes related to antibiotic resistance were identified (Figure S4), including 62 antibiotic resistance genes, 21 antibiotic target genes, and 1 antibiotic biosynthesis gene.

Analysis using the Carbohydrate-Active enZYmes Database (CAZy) identified 145 carbohydrate-active enzymes in the SF416 genome (Figure 3B). These include 33 glycoside hydrolases (GHs), 39 glycosyl transferases (GTs), 4 polysaccharide lyases (PLs), 35 carbohydrate esterases (CEs), 14 auxiliary activities (AAs), and 10 carbohydrate-binding modules (CBMs). Notably, GHs contain two chitinases (EC 3.2.1.14) and six lysozymes type G (EC 3.2.1.17), which are associated with the degradation of fungal and Gram-positive bacterial cell walls; two endoglucanases (EC 3.2.1.4) and two beta-glucosidases (EC 3.2.1.21), which are related to cellulose degradation; and one alpha, alpha-trehalase (EC 3.2.1.28), which is involved in trehalose synthesis (Table 3).

Genomic annotation of *P. aeruginosa* SF416 revealed the presence of several genes associated with environmental adaptability, including genes related to cell motility (*flgABCDEFGHIKL*, *motABY, che*, *mcp*, *efp*, etc.), colonization (*miCDE*), cold shock proteins (*csp*AD), heat shock proteins (*dnaJ*, *yegD*, *hslO*, etc.), and antibiotic resistance (Table S2). Additionally, genes related to plant growth promotion were identified, including 1-aminocyclopropane-1-carboxylate (ACC) deaminase, pyrroloquinoline quinone (PQQ, encoded by *pqqBCDEF*), quinoprotein glucose dehydrogenase (*gcd*), nitrogen metabolism (*nasADE*, *norBC*, *napAB*, *nirS*, etc.), phosphate metabolism (*pstBST*, *phoAB*, etc.), and indole-3-acetic acid (IAA) production (*trp*) (Table S3). In addition, SF416 produced clear zones on extracellular protease detection medium, indicating its ability to produce protease (Figure S5A). These results suggest that *P. aeruginosa* SF416 harbors genes associated with growth-promoting characteristics in plant root environments.

### 3.4. Analysis of Antagonistic Mechanism of P. aeruginosa SF416 Against Xoo

**Figure 4:**
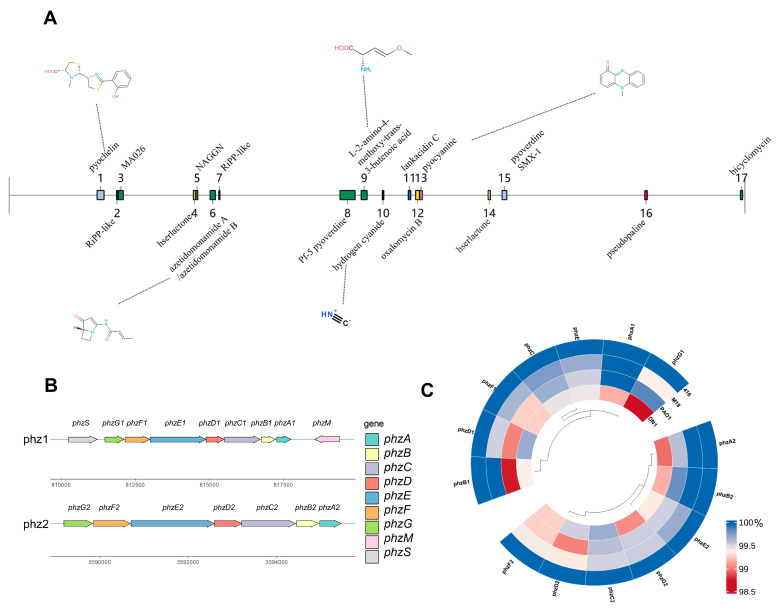
Analysis of secondary metabolites in the SF416 genome. (**A**) Secondary metabolite gene clusters in the SF416 genome predicted using the antiSMASH online tool, and the closest compound structures of each corresponding region to those in the MiBIG database. (**B**) The two sets of phenazine-1-carboxylic acid (PCA) synthesis gene clusters, phz1 (*phzA1*-*G1*) and phz2 (*phzA2*-*G2*), in the genomes of *P*. *aeruginosa* SF416. (**C**) Similarity analysis of phenozine product synthesis-related genes between SF416 and the other three *P*. *aeruginosa* strains.

To explore the antagonistic mechanism of *P*. *aeruginosa* SF416 against *Xoo*, we analyzed the secondary metabolite gene clusters in the genome of SF416 using the antiSMASH online tool. The results reveal that a total of 17 secondary metabolite gene clusters were detected in its genome; among these, 12 known secondary metabolite gene clusters showed similarity to known clusters, including 5 clusters (azetidomonamide A/B, L-2-amino-4-methoxy-trans-3-butenoic acid, hydrogen cyanide, pyocyanine, and pseudopaline) which showed 100% similarity, 1 cluster (pyochelin) with 92% similarity, and 1 cluster (bicyclomycin) with 75% similarity (Figure 4A and Table 4), indicating that SF416 has a high probability of synthesizing these secondary metabolites. The structures of the compounds most similar to those in the MiBIG database are shown in Figure 4A. Notably, these products include various antibacterial substances such as L-2-amino-4-methoxy-trans-3-butenoic acid (AMB), hydrogen cyanide, and bicyclomycin, as well as two siderophores, pyochelin and pyoverdine. These siderophores likely inhibit the growth of pathogens by competing for iron ions in the environment. Our experiments showed that SF416 was able to form an orange halo on chrome azurol S (CAS) agar, demonstrating its ability to produce siderophores (Figure S5B).

Since the Region 13 cluster in the genome of SF416 showed 100% similarity with the known gene cluster coding for Pyocyanin (PYO), which is the derivative of phenazine-1-carboxylic acid (PCA). We further analyzed phenazine compound synthesis gene clusters in the SF416 genome and found that two sets of PCA synthesis gene clusters, phz1 (*phzA1-G1*) and phz2 (*phzA2-G2*), as well as other phenozine product-synthesis-related genes *phzS*, *phzM,* and *phzH,* are present in the genome of SF416 (Figure 4B). Generally, under the action of two sets of PCA synthesis gene clusters, *phzA1-G1* and *phzA2-G2*, chorismate generates PCA, which is transformed into 1-hydroxyphenazine under the action of PhzS, pyocyanin (PYO) under the action of PhzM and PhzS, and phenazine-1-carboxamide (PCN) under the action of PhzH [41]. We compared these genes between SF416 and three *P. aeruginosa* strains PAO1, DN1, and M18, and found that these genes in the SF416 genome share highly similarity (all above 98%) with the ones in the genomes of three *P. aeruginosa* strains, but have the smallest sequence difference with the ones in the genome of *P. aeruginosa* M18 (Figure 4C and Table S4), which is a plant growth-promoting rhizobacterium, indicating that these genes are conserved among different *P. aeruginosa*. In our previous study, PCA exhibited an obvious antagonistic effect against *Xoo* PXO99^A^; therefore, we speculated that the two sets of PCA synthesis gene clusters, *phzA1-G1* and *phzA2-G2*, are responsible for the antagonistic effect of *P*. *aeruginosa* SF416 against *Xoo*.

### 3.5. Comparative Genomic Analysis of P. aeruginosa SF416 with Other Representative P. aeruginosa Strains

We selected four closely related strains, *P. aeruginosa* PAO1, *P. aeruginosa* M18, *P. aeruginosa* DN1, and *P. otitidis* MrB4, for comparative genomic analysis with SF416. A comparison of the genomic features of SF416 with those of the selected reference strains (Table S5) showed that SF416 had the smallest GC content. The whole genome size, GC content, CDS, as well as the number of rRNAs and tRNAs of SF416 were closest to that of the plant growth-promoting rhizobacterium (PGPR) M18, which is also in agreement with the results of the genome-wide evolutionary tree.

**Figure 5:**
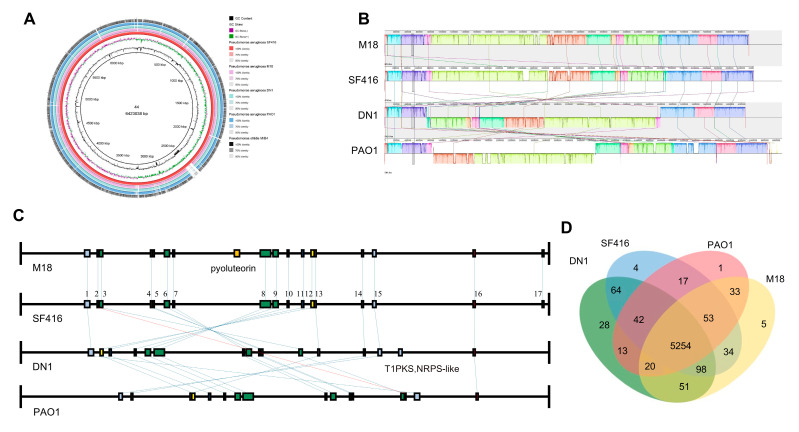
Comparative genomic analysis between SF416 and the other four strains of Pseudomonas strains. (**A**) Circle of similarity plots between SF416 and the other four strains of *Pseudomonas* spp. From the inside out, the first circle represents the scale; the second circle represents the GC content; the third circle represents the GC Skew; the fourth to the eighth circles represent *P. aeruginosa* SF416, M18, DN, PAO1, and *P. otitidis* MrB4, respectively. (**B**) Collinearity comparison of the genomes of the four *P. aeruginosa* strains using *P. aeruginosa* SF416 as the reference genome. (**C**) Comparison of secondary metabolite gene clusters of the four strains predicted using the antiSMASH online tool, with Region1–17 corresponding to the clusters in Table 4. (**D**) Analysis of the straight homologue clusters of the four *P. aeruginosa* strains.

To examine the similarity of the whole genomes, we used BRIG software (v0.95) to create genome circle maps for five strains, with SF416 as the reference strain (Figure 5A). The genome circle maps revealed high similarity among the genomes of the selected *Pseudomonas* strains, with some insertions and deletions. Using Mauve software (v2.4.0), we conducted a synteny analysis of the four *P. aeruginosa* strains, excluding *P. otitidis* MrB4. The synteny analysis showed that the four *P. aeruginosa* strains had many locally collinear blocks, indicating many conserved regions in the genomes of SF416 and the other *P. aeruginosa* strains (Figure 5B). We also found a large segment of chromosome inversion of different lengths in both PAO1 and DN1 strains compared to SF416 and M18, which may be due to the recombination of the *rrnA* and *rrnB* genes [42]. This inversion is also present in different PAO1 sublines and causes phenotypic differences [43]. This may be one of the reasons for the differences between different *P. aeruginosa* strains.

We compared the secondary metabolite gene clusters of SF416 with the other four *P. aeruginosa* strains using antiSMASH online analysis combined with the MiBIG database. The genomes of *P. aeruginosa* SF416, PAO1, M18, DN1, and *P. otitidis* MrB4 were predicted to contain 17, 16, 18, 16, and 10 secondary metabolite gene clusters, respectively (Tables S6–S9). MrB4 had only two relatively conserved regions compared to the other four *P. aeruginosa* strains. The secondary metabolite gene clusters were relatively conserved among the four *P. aeruginosa* strains, with 15 regions (region 1, 3–16) conserved in all four strains, one region (region 2) conserved in SF416, PAO1, and M18, and one region (region 17) conserved only in SF416 and M18 (Figure 5C). The secondary metabolite gene cluster conserved exclusively in SF416 and M18 was predicted to be associated with bicyclomycin, showing 75% similarity. Bicyclomycin is a broad-spectrum antibiotic, which might be a unique mechanism of antibacterial activity for SF416 and M18.

Using the OrthoVenn 3 online tool, we performed an orthologous cluster analysis on the four *P. aeruginosa* strains. The results showed that 5254 orthologous clusters were shared between these strains, with 1, 4, 5, and 28 orthologous clusters being individually assigned to *P. aeruginosa* PAO1, SF416, M18, and DN1 (Figure 5D). There were thirty-four orthologous clusters identified in the SF416 and M18 genomes, of which four were involved in the maintenance of CRISPR repeat elements, two in the salicylic acid catabolic process, two in the auxin catabolic process, two in metal ion binding, and two in oxidoreductase activity (Table S10). Four unique orthologous clusters in SF416 were not annotated in the GO and Swiss-prot in the annotation (Table S11).

## 4. Discussion

In this study, we isolated and identified a strain of *P. aeruginosa*, SF416, and revealed its potential in controlling bacterial blight of rice caused by *Xoo* through the field biocontrol experiment, whole genome sequencing, and functional genes analysis. *P. aeruginosa* SF416 exhibited effective control of bacterial blight of rice in field trials. Whole genome analysis revealed multiple genes in SF416 associated with plant growth promotion and stress resistance, including those involved in IAA synthesis, phosphate solubilization, cellulose degradation, and ACC deaminase activity. Additionally, SF416 possesses the ability to synthesize various antimicrobial substances, such as phenazine antibiotics, siderophores, and hydrogen cyanide, which inhibit pathogen growth through multiple mechanisms. This study preliminarily elucidates the potential multiple mechanisms of SF416 in controlling bacterial blight of rice, providing some evidence for its application in the field of biological control.

Analysis of the *P. aeruginosa* SF416 genome revealed several mechanisms that may promote plant growth, including the synthesis of indole-3-acetic acid (IAA), improved soil phosphorus nutrition, and ACC deaminase activity. SF416 contains the *trpABCDEFGS* genes, indicating its ability to synthesize IAA. IAA is a crucial plant hormone that regulates growth, nutrient absorption, and defense responses [44]. IAA also plays an essential role in root tissue formation, with changes in auxin levels correlating with root growth variations [45]. Phosphorus (P) is vital for plant growth and development, playing a significant role in various physiological and biochemical activities. *P. aeruginosa* SF416 possesses genes (*phoAB*) encoding alkaline phosphatase (ALD), which can convert insoluble organic or inorganic phosphorus into soluble forms available for plant uptake, thereby promoting plant growth. For example, the phosphate-solubilizing *Pseudomonas* strain P34-L improves wheat plant growth by enhancing root structure and soil phosphorus nutrition [46]. The common mechanism in Gram-negative bacteria for solubilizing phosphate involves the release of organic acids that chelate divalent cations (e.g., Ca^2+^) from mineral phosphate complexes, releasing free phosphate for plant uptake. Many Gram-negative bacteria use a pyrroloquinoline quinone (PQQ)-dependent glucose dehydrogenase (GDH) to oxidize glucose periplasmically, producing gluconic acid, which can dissolve mineral phosphate, reduce protozoan predation, and act as an antifungal agent [47]. *P. aeruginosa* SF416 contains genes related to the synthesis of PQQ and quinoprotein glucose dehydrogenase, enabling it to solubilize insoluble soil phosphates. To summarize, these suggest that *P. aeruginosa* SF416 not only enhances plant growth by synthesizing IAA and improving phosphorus availability, but also employs various biochemical pathways such as ACC deaminase activity and PQQ-dependent phosphate solubilization. These mechanisms collectively demonstrate the potential of SF416 as a plant growth-promoting bacterium.

Moreover, as a plant growth-promoting rhizobacterium (PGPR), a key trait of *P. aeruginosa* SF416 is its ability to control ethylene formation via ACC (1-aminocyclopropane-1-carboxylate) deaminase. This enzyme hydrolyzes ACC secreted by plant roots into ammonia and alpha-ketobutyrate, stimulating the extrusion of ACC from roots into the soil [48]. Lowering ACC concentrations in root tissues reduces endogenous ethylene formation, thus promoting plant growth. Reducing ethylene-mediated growth inhibition can also enhance drought tolerance in plants [49,50]. While *P. aeruginosa* SF416 exhibits strong environmental adaptability, making it widely applicable in agricultural production. CARD predictions indicate 62 antibiotic resistance genes in the *P. aeruginosa* SF416 genome, suggesting robust antibiotic resistance. The *P. aeruginosa* SF416 genome also contains multiple genes related to extreme temperature tolerance. Heat shock proteins, which significantly increase in expression under heat stress, protect cells from various adverse conditions [51]. *P. aeruginosa* SF416 has multiple heat shock protein genes (*hslO*, *dnaJ*). The *hslO* gene controls the formation of siderophores in *Pseudomonas* SN15-2, while *dnaJ* influences the motility of the strain [52]. In summary, *P. aeruginosa* SF416 shows great potential in enhancing nutrient uptake, improving stress tolerance, and regulating ethylene production. Its strong adaptability and antibiotic resistance make it a viable candidate for use in a wide range of agricultural settings.

While SF416 exhibits strong environmental adaptability, making it widely applicable in agricultural production. CARD predictions indicate 62 antibiotic resistance genes in the SF416 genome, suggesting robust antibiotic resistance. The SF416 genome also contains multiple genes related to extreme temperature tolerance. Heat shock proteins, which significantly increase in expression under heat stress, protect cells from various adverse conditions [51]. SF416 has multiple heat shock protein genes (*hslO*, *dnaJ*). At the same time, the *hslO* gene controls the formation of siderophores in *Pseudomonas* SN15-2, while *dnaJ* influences the motility of the strain [52].

Endophytic bacteria capable of degrading cellulose can use agricultural waste as a carbon source and promote plant health through their antibacterial properties. *P. aeruginosa* SF416 likely utilizes enzymes related to cellulose degradation, such as endoglucanase (EC 3.2.1.4) and beta-glucosidase (EC 3.2.1.21), to better utilize cellulose in the rhizosphere, enhancing its colonization capability [53]. *P. aeruginosa* SF416 can also form biofilms using alginate, psl, and pel polysaccharides conserved in *P. aeruginosa*, aiding effective colonization on soil or plant surfaces and occupying ecological niches [54]. EPSs play critical roles in bacterial interactions with the environment, forming organic molecules through the polymerization of carbohydrates, proteins, and humic substances [55]. Under adverse conditions, the synthesis of EPSs containing multiple polysaccharides can stabilize cell structures and protect them from stress. It is reported that alginate can replace water molecules around macromolecules to protect cell enzymes and stabilize cell membranes under stress conditions [56]. Studies have shown that EPS in *P. aeruginosa* functions differently under various conditions: under low salt stress, EPS on the surface of *P. aeruginosa* PF23 primarily serves as a biocontrol agent, while under high salt stress, EPS acts as an osmotic protector. In high-salt stress conditions, *P. aeruginosa* PF23 produces a large number of EPSs, which aggregate around the cells, significantly enhancing bacterial cell survival under salt stress [57]. Therefore, the presence of these resistance genes significantly enhances *P. aeruginosa* SF416’s stress tolerance, thus improving its potential for agricultural applications.

The primary mechanisms by which biocontrol bacteria exert their antimicrobial effects include direct secretion of antimicrobial substances, competition for nutrients and space, and induction of plant systemic resistance. *P. aeruginosa* SF416 can produce various antimicrobial substances like phenazine antibiotics (1-hydroxyphenazine, Phenazine-1-carboxylic acid, pyocyanin) [58], siderophores (pyoverdine and pyochelin), cell wall-degrading enzymes, HCN, and rhamnolipids [59]. AntiSMASH predictions indicated that *P. aeruginosa* SF416 can produce two siderophores (pyochelin and pyoverdine). Siderophores chelate available iron in the soil, reducing the iron available to plant pathogens and thus inhibiting their growth. Beyond competition, siderophores can also induce plant systemic resistance, preventing pathogen infection [60]. SF416 possesses two complete *phz* gene clusters and downstream modification genes, suggesting its ability to synthesize phenazine antibiotics. Bicyclomycin, a broad-spectrum antibiotic, selectively inhibits the bacterial transcription termination factor Rho [61]. Some studies have found that homologous gene clusters for bicyclomycin are widely present and have achieved heterologous expression in *P. aeruginosa* [62]. AMB (L-2-amino-4-methoxy-trans-3-butenoic acid), another antibiotic produced by *P. aeruginosa* biocontrol strains, strongly inhibits *Erwinia amylovora* and interferes with the germination of grass weed seeds [63]. *P. aeruginosa* SF416 also contains hydrogen cyanide (HCN) synthesis genes (*hcnABC*). HCN is a known plant pathogen inhibitor, and bacterial HCN can induce plant resistance [64]. In short, *P. aeruginosa* SF416 employs multiple biocontrol mechanisms. Its ability to compete for resources and induce plant systemic resistance further enhances its role as an effective biocontrol agent.

The CAZy analysis indicates that the SF416 genome contains six lysozyme type G genes (EC 3.2.1.17) and two chitinase genes (EC 3.2.1.14). Chitin is a primary component of fungal cell walls, and chitinases act on the β-1,4-glycosidic bonds of chitin. Studies have shown that chitinases not only defend against many fungal pathogens, but are also associated with induced resistance in crops [65]. Chitinase (EC 3.2.1.14) belongs to the pathogenesis-related protein (PR protein) family. Chitinase hydrolyze fungal cell walls, releasing chitin and glucan fragments, which are oligosaccharides with elicitor activity that induce defense responses in host plants [66]. Rhamnolipids are low-molecular-weight glycolipids primarily secreted by *Pseudomonas* species, consisting of one or two rhamnose molecules linked to one or two β-hydroxy fatty acids. Due to their antibacterial properties and ability to induce plant defense responses, rhamnolipids have been extensively studied [67]. It has been reported that rhamnolipids produced by *P. aeruginosa* DR1 can inhibit the growth of various plant pathogens, such as *Sclerotium rolfsii, Fusarium oxysporium, Phytophthora nicotianae*, and *Macrophomina phaseolina* [68]. These analyses indicated that *P. aeruginosa* SF416 exhibits antagonistic activity against a wide range of plant pathogenic bacteria and fungi through multiple mechanisms, including the secretion of antimicrobial substances, competition with pathogens, and the induction of systemic resistance in plants.

In summary, we identified *P*. *aeruginosa* SF416 and demonstrated that it is a potential biocontrol agent for bacterial blight of rice. This study not only finds a new biocontrol resource against *Xoo*, but also lays a foundation for the further biological control of bacterial disease of rice in the fields.

## Figures and Tables

**Table 1:** Inhibition effect of SF416 against various plant pathogenic bacteria and fungi.

Plant-Pathogenic Bacteria	Inhibitory Diameter (mm)	Relative Inhibition (%)
*X. oryzae* pv. *oryzae* PXO99^A^	52.53 ± 3.11	84.74 ± 0.88
*X. oryzae* pv. *oryzicola* RS105	39.23 ± 0.25	79.61 ± 0.13
*X. citri* subsp. *citri*	16.57 ± 0.32	51.67 ± 0.27
*X. campestris* pv. *musacearum*	18.8 ± 1.44	57.27 ± 3.38
*X. campestris* pv. *malvacearum*	18.67 ± 1.53	56.95 ± 3.62
*X. axonopodis* pv. *glycines*	27.83 ± 0.29	71.26 ± 0.3
*X. campestris* pv. *vesicatoria*	25.33 ± 0.58	68.41 ± 0.71
*X. campestris* pv. *phaseoli*	48.5 ± 1.73	83.49 ± 0.6
*X. axonopodis* pv. *vignicola*	22.4 ± 0.53	64.27 ± 0.83
*X. axonopodis* pv. *vasculorum*	19.87 ± 0.81	59.69 ± 1.62
*X. axonopodis* pv. *allii*	16.07 ± 1.79	49.76 ± 5.99
*X. campestris* pv. *juglandis*	15.2 ± 0.96	47.23 ± 3.24
*Acidovorax citrulli*	17.47 ± 0.65	54.16 ± 1.71
*P. syringae* pv. *tomato DC3000*	20.33 ± 0.58	60.63 ± 1.1
**Plant-Pathogenic Fungi**	**Growth Radius (mm)**	**Relative Inhibition (%)**
*Magnaporthe oryzae*	12.33 ± 0.82	79.39 ± 2.15
*Fusarium oxysporium*	10.67 ± 0.52	71.93 ± 1.36
*Botrytis cinerea*	12.83 ± 2.04	64.84 ± 5.59
*Fusarium graminearum*	10.83 ± 1.03	69.48 ± 2.91
*Phytophthora capsici*	5.5 ± 1.22	83.82 ± 3.6
*Colletotrichum gloeosporioides*	9.23 ± 0.5	74.35 ± 1.39

**Table 2:** General characterization of the *P*. *aeruginosa* SF416 genome.

Attribute	Value
Genome size	6,423,038 bp
GC content (%)	66.23%
ORF number	5785
ORF average length	976.74 bp
ORF/Genome (coding percentage)	87.97%
GC content in ORF region	67.07%
GC content in intergenetic region	60.14%
rRNA	4, 4, 4 (5S, 16S, 23S)
RNA	65
ncRNA	71
CRISPR Array	6
Prophage	9
Genomics Islands	33
CARD	78
CAZyme	145

**Table 3:** Partial GHs in the genome of *P*. *aeruginosa* SF416.

Class_Definition	Start	End	Strand	Family
chitinase (EC 3.2.1.14)	3105281	3106732	+	GH18
chitinase (EC 3.2.1.14)	717666	718295	+	GH19
lysozyme type G (EC 3.2.1.17)	1309786	1311258	−	GH23
lysozyme type G (EC 3.2.1.17)	2116706	2118634	−	GH23
lysozyme type G (EC 3.2.1.17)	2282185	2283615	+	GH23
lysozyme type G (EC 3.2.1.17)	3182477	3183073	−	GH23
lysozyme type G (EC 3.2.1.17)	3692961	3694508	+	GH23
lysozyme type G (EC 3.2.1.17)	6200389	6200982	−	GH23
endoglucanase (EC 3.2.1.4)	2046560	2047465	+	GH74
endoglucanase (EC 3.2.1.4)	4031350	4032456	−	GH74
beta-glucosidase (EC 3.2.1.21)	2133851	2134834	+	GH3
beta-glucosidase (EC 3.2.1.21)	3795750	3798044	+	GH3
alpha,alpha-trehalase (EC 3.2.1.28)	2939027	2940640	−	GH37

**Table 4:** The secondary metabolism gene clusters in the genome of *P*. *aeruginosa* SF416 predicted using AntiSMASH.

Region	Type	Location	Most Similar Known Cluster	Similarity
Region 1	NRPS, phenazine	766,489–827,772	pyochelin (NRP)	92%
Region 2	RiPP-like	936,167–946,997	unknown	ND
Region 3	NRPS-like	953,164–996,139	MA026(NRP)	5%
Region 4	hserlactone	1,606,956–1,627,561	unknown	ND
Region 5	NAGGN	1,632,061–1,646,821	unknown	ND
Region 6	NRPS	1,753,162–1,800,220	azetidomonamide A/B (NRP)	100%
Region 7	RiPP-like	1,828,981–1,839,835	unknown	ND
Region 8	NRPS, NRP-metallophore	2,887,014–3,019,644	Pf-5 pyoverdine (NRP)	25%
Region 9	NRPS	3,072,171–3,124,451	L-2-amino-4-methoxy-trans-3-butenoic acid (NRP)	100%
Region 10	hydrogen cyanide	3,257,196–3,270,157	hydrogen cyanide	100%
Region 11	redox cofactor	3,483,660–3,505,804	lankacidin C (NRP+Polyketide)	13%
Region 12	thiopeptide	3,548,102–3,581,105	oxalomycin B (NRP+Polyketide)	6%
Region 13	phenazine	3,584,452–3,605,464	pyocyanine	100%
Region 14	hserlactone	4,180,614–4,201,219	unknown	ND
Region 15	NRPS-like, betalactone	4,302,567–4,344,387	pyoverdine SMX-1(NRP)	12%
Region 16	opine-like-metallophore	5,548,699–5,570,788	pseudopaline	100%
Region 17	CDPS	6,382,943–6,403,677	bicyclomycin	75%

NRPS = nonribosomal peptide synthases; NRP-metallophore = Non-ribosomal peptide metallophore; redox-cofactor = Redox-cofactors such as PQQ; RiPP = Other unspecified ribosomally synthesised and post-translationally modified peptide product; hserlactone = Homoserine lactone; NAGGN = N-acetylglutaminylglutamine amide; opine-like-metallophore = Opine-like zincophores like staphylopine; ND indicates that a similar gene cluster was not detected in the antiSMASH database.

## Data Availability

The sequencing data for this project are available on the National Center for Biotechnology Information (NCBI) repository under BioProject: PRJNA970529; BioSample: SAMN35005130; Genomes: CP125288.

## Supplementary Materials

The following supporting information can be downloaded at: https://www.mdpi.com/article/10.3390/microorganisms12112263/s1, Figure S1. The morphology of SF416 on NA medium and under 100× light microscope; Figure S2. Inhibitory effect of SF416 against some plant pathogenic bacteria; Figure S3. Inhibitory effect of SF416 against some plant pathogenic fungi; Figure S4: Antibiotic-related genes in the SF416 genome; Figure S5. SF416 can secrete cellular protease and siderophores. Table S1. Inhibition effect of the cell-free supernatant of SF416 against *Xoo* PXO99^A^ and *Xoc* RS105; Table S2. Genes related to environmental adaptation in the genome of *P*. *aeruginosa* SF416; Table S3. Genes related to plant growth promotion in the genome of *P*. *aeruginosa* SF416; Table S4. Similarity analysis of phenozine product synthesis related genes between SF416 and other three *P*. *aeruginosa* strain; Table S5. Comparison of general genomic characteristics between *P*. *aeruginosa* SF416 and other four *Pseudomonas* strains; Table S6. The secondary metabolism gene clusters in the genome of *P*. *aeruginosa* M18 predicted by AntiSMASH; Table S7. The secondary metabolism gene clusters in the genome of *P*. *aeruginosa* PAO1 predicted by AntiSMASH; Table S8. The secondary metabolism gene clusters in the genome of *P*. *aeruginosa* DN1 predicted by AntiSMASH; Table S9. The secondary metabolism gene clusters in the genome of *P*. *aeruginosa* MR48 predicted by AntiSMASH; Table S10. The same orthologous clusters identified in the *P*. *aeruginosa* SF416 and M18 genomes; Table S11. The unique orthologous clusters in the *P*. *aeruginosa* SF416 genome.
