# Supplementary material for: Whole Genome Sequencing and Comparative Genomic Analysis of Pseudomonas aeruginosa SF416, a Potential Broad-Spectrum Biocontrol Agent Against Xanthomonas oryzae pv. oryzae
Source: Microorganisms. 2024 Nov 8;12(11):2263. doi: 10.3390/microorganisms12112263 (PMC11596105; doi:10.3390/microorganisms12112263)
Supplement: Supplementary file 1 [file microorganisms-12-02263-s001.zip › Supplementary Figures.pdf]

Supplementary Figures

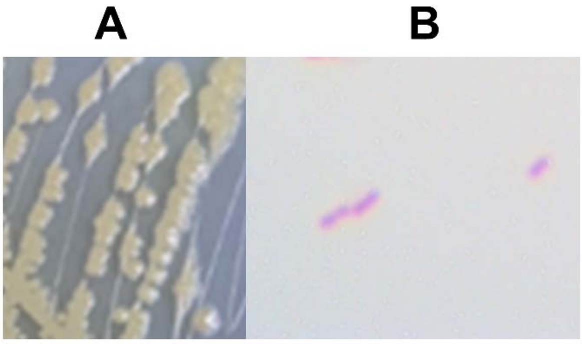

Figure S1. The morphology of SF416 on NA medium(A) and under 100X light microscope (B).

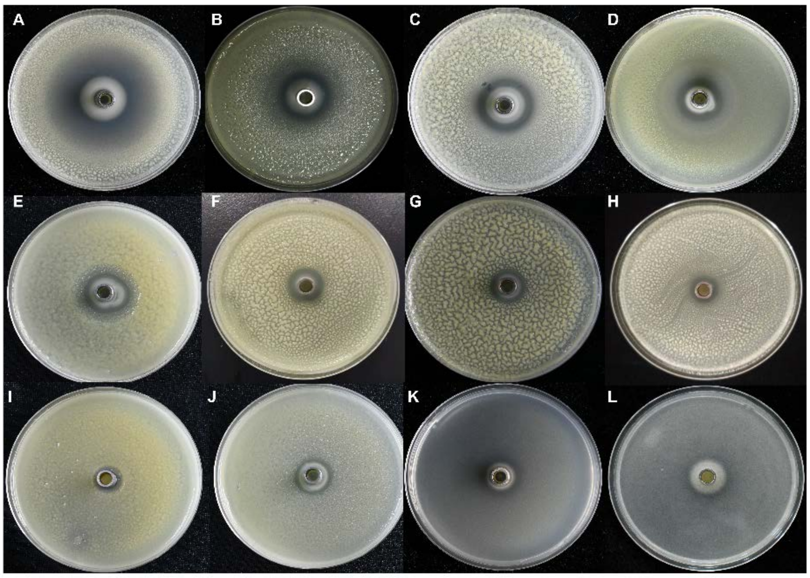

Figure S2. Inhibitory effect of SF416 against some plant pathogenic bacteria. (A) *X. citri* subsp. *citri*; (B) *X. campestris* pv. *musacearum*; (C) *X. campestris* pv. *malvacearum*; (D) *X. axonopodis* pv. *glycines*; (E) *X. campestris* pv. *vesicatoria*; (F) *X. campestris* pv. *phaseoli*; (G) *X. axonopodis* pv. *vignicola*; (H) *X. axonopodis* pv. *vasculorum*; (I) *X. axonopodis* pv. *allii*; (J) *X. campestris* pv. *juglandis*; (K) *Acidovorax citrulli*; (L) *Pseudomonas syringae* pv. *tomato* DC3000

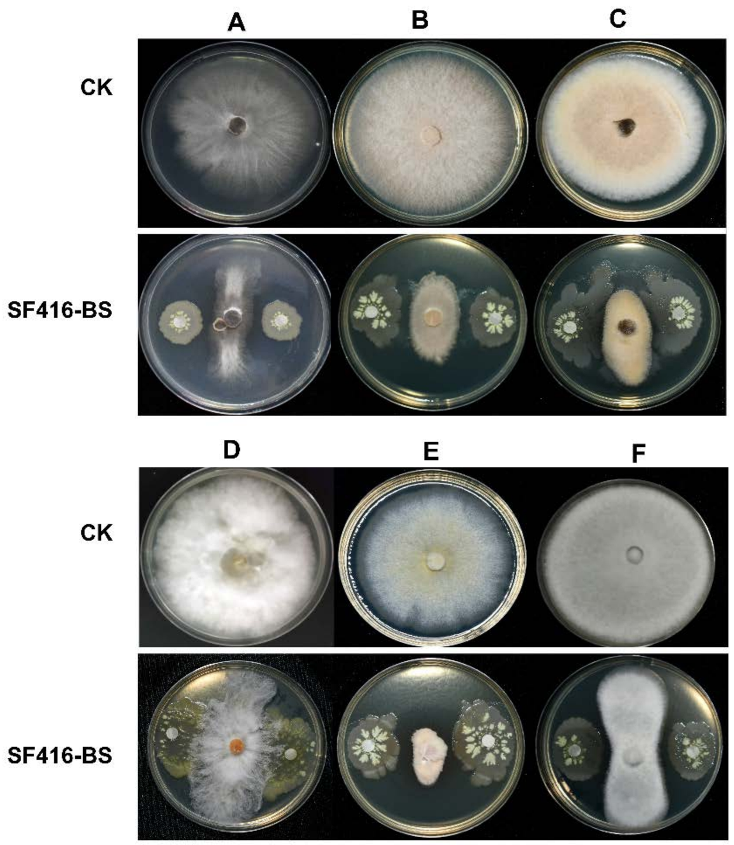

Figure S3. Inhibitory effect of SF416 against some plant pathogenic fungi. (A) *Magnaporthe oryzae*; (B) *Fusarium oxysporium*; (C) *Botrytis cinerea*; (D) *Fusarium graminearum*; (E) *Phytophthora capsica*; (C) *Colletotrichum gloeosporioides.*

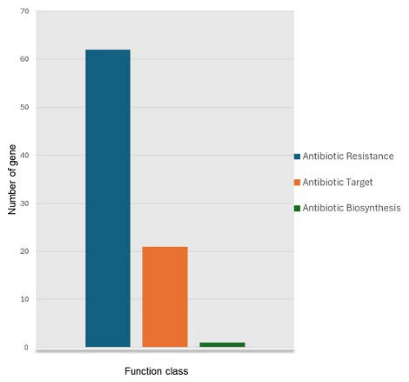

Figure S4: Antibiotic-related genes in the SF416 genome.

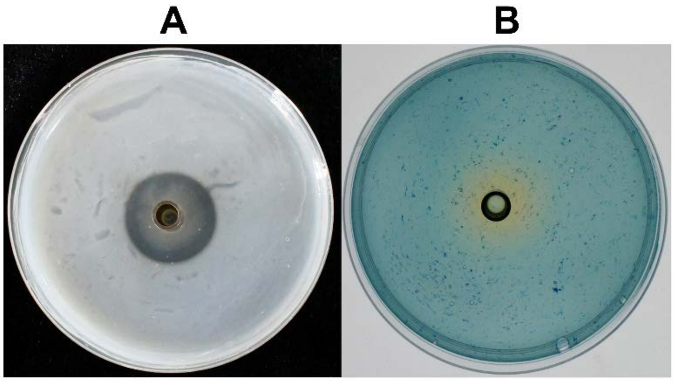

Figure S5. SF416 can secrete cellular protease (A) and siderophores (B).
