# Supplementary material for: Whole Genome Sequencing and Comparative Genomic Analysis of Pseudomonas aeruginosa SF416, a Potential Broad-Spectrum Biocontrol Agent Against Xanthomonas oryzae pv. oryzae
Source: Microorganisms. 2024 Nov 8;12(11):2263. doi: 10.3390/microorganisms12112263 (PMC11596105; doi:10.3390/microorganisms12112263)
Supplement: Supplementary file 1 [file microorganisms-12-02263-s001.zip › Supplementary Table.pdf]

Supplementary Tables

Table S1 Inhibition effect of the cell-free supernatant of SF416 against *Xoo* PXO99^{A} and *Xoc* RS105

Pathogen	Concentration of CFS	Inhibitory diameter	Inhibitory rate
Xoo PXO99^{A}	10X	33.67±1.89	76.16%±1.39%
	5X	28.33±0.47	71.76%±0.46%
	1X	12.67±1.25	36.20%±6.56%
Xoc RS105	10X	21.33±0.47	62.48%±0.82%
	5X	16.67±1.89	51.32%±5.99%
	1X	--	--

-- indicates no inhibition effect was observed.

Table S2 Genes related to environmental adaptation in the genome of *P. aeruginosa* SF416

	Genesymbol	Start	End	SWISSID	SWISS_Description
Flagellation	*flgA*	1748118	1748816	Q56892	Flagella basal body P-ring formation protein FlgA
	flgL	4469465	4470784	P16326	Flagellar hook-associated protein 3
	flgK	4470810	4472861	P33235	Flagellar hook-associated protein 1
	flgJ	4472880	4474082	Q9I4P4	Peptidoglycan hydrolase FlgJ
	flgI	4474093	4475202	Q9I4P5	Flagellar P-ring protein
	flgH	4475214	4475909	Q9I4P6	Flagellar L-ring protein
	flgG	4475955	4476740	P0A1J3	Flagellar basal-body rod protein FlgG
	flgF	4476787	4477536	P75938	Flagellar basal-body rod protein FlgF
	flgE	4477754	4479142	P75937	Flagellar hook protein FlgE
	flgD	4479170	4479883	P0A1I9	Basal-body rod modification protein FlgD
	flgC	4479896	4480336	P0A1I7	Flagellar basal-body rod protein FlgC
	flgB	4480342	4480749	Q8GLP8	Flagellar basal body rod protein FlgB
Motility	*motB*	115507	116856	P28612	Motility protein B
	motY	1583438	1584382	Q9S3P9	Sodium-type flagellar protein MotY
	motA	4159206	4159946	P28611	Motility protein A
	motB	5690869	5691912	P0AF06	Motility protein B
	motA	5691932	5692783	P55891	Motility protein A
	pilT	454443	455477	P24559	Type IV pilus retractation ATPase PilT
	pilU	455655	456803	G3XCX3	Type IV pilus ATPase PilU
	swrC	199997	203044	O31501	Swarming motility protein SwrC
	rssA	4820105	4821424	Q8GP19	Swarming motility regulation sensor protein RssA
Chemotaxis	*cheB2*	217487	218536	Q9I6V9	Protein-glutamate methylesterase/protein-glutamine glutaminase 2
	cheD	218556	219158	A4G4P0	Probable chemoreceptor glutamine deamidase CheD
	cheR2	219164	220006	Q9I6V7	Chemotaxis protein methyltransferase 2
	cheW	222151	222540	P21821	Chemotaxis protein CheW
	cheA	222623	224530	P21813	Chemotaxis protein CheA
	cheY	224570	224935	Q9FAD7	Chemotaxis protein CheY
	cheY	319911	321110	P0A2D5	Chemotaxis protein CheY
	cheR	471113	471988	O51069	Chemotaxis protein methyltransferase
	cheAY	472000	479418	O25153	Sensor histidine kinase CheAY
	cheB3	479441	480442	P62640	Protein-glutamate methylesterase/protein-glutamine glutaminase 3
	cheW	1380274	1380789	P21821	Chemotaxis protein CheW
	cheAY	1382733	1385042	O25153	Sensor histidine kinase CheAY
	cheB3	1385063	1386046	Q9HXT8	Protein-glutamate methylesterase/protein-glutamine glutaminase 3
	cheV1	1748948	1749880	O24864	Chemotaxis protein CheV1
	cheR1	1749984	1750781	O87131	Chemotaxis protein methyltransferase 1
	cheW	4155913	4156392	Q52881	Chemotaxis protein CheW
	cheB1	4160035	4161141	O87125	Protein-glutamate methylesterase/protein-glutamine glutaminase 1
	cheAY	4161195	4163453	O25153	Sensor histidine kinase CheAY
	cheZ	4163654	4164442	Q02JU1	Protein phosphatase CheZ
	cheY	4164462	4164848	Q51455	Chemotaxis protein CheY
	cheY1	5053189	5054289	P71403	Chemotaxis protein CheY1
	cheY	6181037	6181939	Q51455	Chemotaxis protein CheY

	mcpH	215233	217224	Q88R14	Methyl-accepting chemotaxis protein McpH
	mcpB	1378641	1380269	P39215	Methyl-accepting chemotaxis protein McpB
	mcpQ	2279524	2280996	Q88D09	Methyl-accepting chemotaxis protein McpQ
	mcpQ	2363104	2364699	Q88D09	Methyl-accepting chemotaxis protein McpQ
	mcpU	2600946	2603090	Q88NI1	Methyl-accepting chemotaxis protein McpU
	mcpQ	5189289	5191310	Q88D09	Methyl-accepting chemotaxis protein McpQ
	mcpU	5329457	5331595	Q88NI1	Methyl-accepting chemotaxis protein McpU
	mcpQ	5841801	5843744	Q88D09	Methyl-accepting chemotaxis protein McpQ
Colonization	*minE*	1878045	1878299	Q9HYZ5	Cell division topological specificity factor
	minD	1878296	1879111	P0AEZ6	Septum site-determining protein MinD
	minC	1879173	1879964	Q9HYZ7	Probable septum site-determining protein MinC
Cold shock protein	*cspA*	531276	531485	P95459	Major cold shock protein CspA
	cspA	1856853	1857062	P95459	Major cold shock protein CspA
	cspD	2642439	2642711	Q9KSW4	Cold shock-like protein CspD
	cspA	4388668	4388877	P95459	Major cold shock protein CspA
	cspA	4597721	4598335	P95459	Major cold shock protein CspA
Heat shock protein	*yegD*	2432366	2433877	P36928	Uncharacterized chaperone protein YegD
	yegD	5603088	5604353	P36928	Uncharacterized chaperone protein YegD
	ibpB	1993559	1994008	P96193	16 kDa heat shock protein B
	hchA	4417724	4418599	Q02IV5	Protein/nucleic acid deglycase HchA
	hslR	5997603	5998004	P0ACG8	Heat shock protein 15
	htpG	4489477	4491318	Q8KE61	Chaperone protein HtpG
	dnaJ	5474947	5476080	Q9HV44	Chaperone protein DnaJ
	hslO	5995756	5996649	Q9HTZ6	33 kDa chaperonin
	dnaK	5476196	5478109	Q9HV43	Chaperone protein DnaK

Table S3 Genes related to plant growth promotion in the genome of *P. aeruginosa* SF416

	Genesymbol	Start	End	Strand	SWISSID	SWISS_Description
phosphate	*phoN*	237632	238357	+	Q934J6	Non-specific acid phosphatase OS
metabolism	*phoD*	1153885	1155438	+	P42251	Alkaline phosphatase D OS
	phoA	1819780	1821168	-	Q02QC9	Alkaline phosphatase H OS
	phoQ	4366942	4368288	-	Q9I4F8	Two-component sensor PhoQ OS
	phoP	4368285	4368962	-	Q9I4F9	Two-component response regulator PhoP OS
	phoA2	4894333	4895511	-	K4LAH1	Alkaline phosphatase L OS
	phoB	6176502	6177140	+	P23620	Phosphate regulon transcriptional regulatory protein PhoB
	phoR	6177225	6178544	+	P23621	Phosphate regulon sensor protein PhoR
	phoU	6182058	6182786	-	Q51547	Phosphate-specific transport system accessory protein PhoU homolog
	pstS	2727536	2728930	-	P0DMR4	Phosphate-binding protein PstS
	pstB	6182882	6183715	-	Q51546	Phosphate import ATP-binding protein PstB
	pstS	6187883	6188854	-	P0DMR4	Phosphate-binding protein PstS
IAA	*trpA*	48576	49382	-	Q02V45	Tryptophan synthase alpha chain
synthesis	*trpB*	49379	50587	-	P07345	Tryptophan synthase beta chain
	trpI	50691	51578	+	P11720	HTH-type transcriptional regulator TrpI
	trpE	700642	702120	+	P20580	Anthranilate synthase component 1
	trpG	719256	719861	+	P20576	Anthranilate synthase component 2
	trpD	719863	720912	+	P20574	Anthranilate phosphoribosyltransferase
	trpC	720909	721745	+	B7V609	Indole-3-glycerol phosphate synthase
	trpF	2008248	2008883	+	Q59649	N-(5'-phosphoribosyl)anthranilate isomerase
	trpI	2213991	2214917	-	P11720	HTH-type transcriptional regulator TrpI
	trpS	5099275	5100621	-	Q9HVX6	Tryptophan--tRNA ligase
nitrogen	*nirS*	595247	596953	-	P24474	Nitrite reductase
metabolism	*norC*	598868	599308	+	Q59646	Nitric oxide reductase subunit C
	norB	599311	600705	+	Q59647	Nitric oxide reductase subunit B
	narL	1187348	1188007	-	P0AF31	Nitrate/nitrite response regulator protein NarL
	narX	1188004	1189872	-	P0AFA4	Nitrate/nitrite sensor protein NarX
	narK1	1190086	1191342	+	Q9RA46	Probable nitrate/nitrite antiporter NarK1
	narK	1191354	1192760	+	P10903	Nitrate/nitrite transporter NarK
	narG	1192836	1196621	+	P09152	Respiratory nitrate reductase 1 alpha chain
	narY	1196633	1198174	+	P19318	Respiratory nitrate reductase 2 beta chain

	narW	1198180	1198920	+	P19317	Probable nitrate reductase molybdenum cofactor assembly chaperone NarW
	narV	1198923	1199606	+	P0AF32	Respiratory nitrate reductase 2 gamma chain
	nasA	3732374	3733585	+	P42432	Nitrate transporter
	nasD	3735523	3737973	+	P42435	Nitrite reductase [NAD(P)H]
	nasE	3738026	3738352	+	P42436	Assimilatory nitrite reductase [NAD(P)H] small subunit
	narB	3738379	3741105	+	P39458	Nitrate reductase
	napA	4370932	4373340	+	B7UX15	Periplasmic nitrate reductase
	napB	4373351	4373842	+	P39186	Periplasmic nitrate reductase, electron transfer subunit
	nrtA	3068640	3069764	+	Q44292	Nitrate/nitrite binding protein NrtA
	nrtC	3729292	3730500	+	P73450	Nitrate import ATP-binding protein NrtC
pyrroloquinoline quinone	*pqqE*	3493660	3494772	-	Q9I2C0	PqqA peptide cyclase
	pqqD	3494777	3495055	-	Q9I2C1	PqqA binding protein
	pqqC	3495052	3495789	-	Q9I2C2	Pyrroloquinoline-quinone synthase
	pqqB	3495814	3496728	-	Q9I2C3	Coenzyme PQQ synthesis protein B
	pqqF	3511018	3513303	-	Q9I2D2	Coenzyme PQQ synthesis protein F
quinoprotein glucose dehydrogenase	*gdhB*	2061292	2066154	+	Q9HZE0	NAD-specific glutamate dehydrogenase
	gdhA	3113144	3115555	+	P05465	Quinoprotein glucose dehydrogenase A
ACC deaminase	*PH0054*	2033781	2034680	+	O57809	Putative 1-aminocyclopropane-1-carboxylate deaminase

Table S4 Similarity analysis of phenozine product synthesis related genes between SF416 and other three *P. aeruginosa* strain.

Gene	Similarity% with PAO1	Similarity% with DN1	Similarity% with M18
phzA1	100	99.18	100
phzB1	98.77	99.39	100
phzC1	99.67	99.43	99.75
phzD1	99.04	99.68	99.52
phzE1	99.52	99.42	99.68
phzF1	99.28	99.28	99.64
phzG1	99.84	98.76	99.38
phzA2	99.59	98.98	100
phzB2	99.8	99.18	100
phzC2	99.59	99.67	99.51
PHZD2	99.04	99.52	99.36
phzE2	99.52	99.36	99.68
phzF2	99.28	99.28	99.4
phzG2	99.53	99.07	99.53

Table S5 Comparison of general genomic characteristics between *P. aeruginosa* SF416 and other four *Pseudomonas* strains

	P. otitidis	*P. aeruginosa*	*P. aeruginosa*	*P. aeruginosa*	*P. aeruginosa*
	**MrB4**	**M18**	**DN1**	**PAO1**	**SF416**
Genome size (Mb)	6.089	6.328	6.642	6.264	6.423
GC content (%)	67.0	66.5	66.5	66.6	66.2
Protein coding sequences (CDS)	5595	5736	6071	5572	5785
rRNA genes	12	12	12	13	12
tRNA genes	74	64	63	63	65

Table S6 The secondary metabolism gene clusters in the genome of *P. aeruginosa* M18 predicted by AntiSMASH

Region	Type	From	To	Most similar known cluster(Type)	Similarity
Region 1	NRP-metallophore, NRPS, phenazine	744,043	805,384	Pyochelin(NRP)	92%
Region 2	RiPP-like	913,997	924,827		
Region 3	NRPS-like	931,060	974,035	MA026(NRP)	5%
Region 4	hserlactone	1,628,943	1,649,548		
Region 5	NAGGN	1,652,684	1,667,444		
Region 6	NRPS	1,770,299	1,817,357	azetidomonamide A/azetidomonamide B(NRP)	100%
Region 7	RiPP-like	1,846,165	1,857,019		
Region 8	T3PKS,T1PKS	2,564,181	2,622,680	pyoluteorin(Polyketide)	100%
Region 9	NRPS,NRP-metallophore	2,842,748	2,974,605	Pf-5 pyoverdine(NRP)	24%
Region 10	NRPS	3,026,266	3,078,546	L-2-amino-4-methoxy-trans-3-butenoic acid(NRP)	100%
Region 11	hydrogen-cyanide	3,164,994	3,177,955	hydrogen cyanide(Other)	100%
Region 12	redox-cofactor	3,400,595	3,422,739	lankacidin C(NRP+Polyketide)	13%
Region 13	thiopeptide	3,464,620	3,497,623	oxalomycin B(NRP+Polyketide)	6%
Region 14	phenazine	3,499,802	3,520,814	pyocyanine (Other)	100%
Region 15	hserlactone	4,096,618	4,117,223		
Region 16	NRPS-like,betalactone	4,215,172	4,256,992	pyoverdine SMX-1(NRP)	12%
Region 17	opine-like-metallophore	5,466,982	5,489,071	pseudopaline(Other)	100%
Region 18	CDPS	6,288,140	6,308,874	bicyclomycin(Other:tRNA-derived)	75%

Table S7. The secondary metabolism gene clusters in the genome of *P. aeruginosa* PAO1 predicted by AntiSMASH

Region	Type	From	To	Most similar known cluster(Type)	Similarity
Region 1	NRPS-like,betalactone	1,302,253	1,344,073	pyoverdine SMX-1(NRP)	12%
Region 2	hserlactone	1,549,254	1,569,859		
Region 3	phenazine	2,060,685	2,081,697	pyocyanine (Other)	100%
Region 4	thiopeptide	2,083,444	2,116,447	oxalomycin B(NRP+Polyketide)	6%
Region 5	redox-cofactor	2,162,913	2,185,057	lankacidin C(NRP+Polyketide)	13%
Region 6	hydrogen-cyanide	2,407,546	2,420,507	hydrogen cyanide(Other)	100%
Region 7	NRPS	2,512,669	2,564,946	L-2-amino-4-methoxy-trans-3-butenoic acid(NRP)	100%
Region 8	NRP-metallophore,NRPS	2,618,928	2,740,694	Pf-5 pyoverdine(NRP)	23%
Region 9	RiPP-like	3,670,894	3,681,748		
Region 10	NRPS	3,710,557	3,757,615	azetidomonamide A/azetidomonamide B(NRP)	100%
Region 11	NAGGN	3,860,897	3,875,657		
Region 12	hserlactone	3,879,139	3,899,744		
Region 13	NRPS-like	4,535,254	4,578,229	MA026(NRP)	5%
Region 14	RiPP-like	4,584,491	4,595,321		
Region 15	phenazine,NRP-metallophore,NRPS	4,703,796	4,765,124	pyochelin(NRP)	
Region 16	opine-like-metallophore	5,415,597	5,437,686	Pseudopaline(Other)	12%

Table S8. The secondary metabolism gene clusters in the genome of *P. aeruginosa* DN1 predicted by AntiSMASH

Region	Type	From	To	Most similar known cluster(Type)	Similarity
Region 1	NRP-metallophore,NRPS,phenazine	753,010	814,365	Pyochelin(NRP)	92%
Region 2	thiopeptide	856,503	889,506	oxalomycin B(NRP+Polyketide)	6%
Region 3	redox-cofactor	932,813	954,957	lankacidin C(NRP+Polyketide)	13%
Region 4	hydrogen-cyanide	1,161,729	1,174,690	hydrogen cyanide(Other)	100%
Region 5	NRPS	1,293,424	1,345,703	L-2-amino-4-methoxy-trans-3-butenoic acid(NRP)	100%
Region 6	NRP-metallophore,NRPS	1,391,733	1,514,597	Pf-5 pyoverdine(NRP)	23%
Region 7	RiPP-like	2,574,590	2,585,444		
Region 8	NRPS	2,614,166	2,661,224	azetidomonamide A/azetidomonamide B(NRP)	100%
Region 9	NAGGN	2,768,318	2,783,078		
Region 10	hserlactone	2,785,811	2,806,416		
Region 11	NRPS-like	3,458,192	3,501,167	MA026(NRP)	5%
Region 12	phenazine	3,658,640	3,679,652	pyocyanine (Other)	100%
Region 13	hserlactone	4,172,466	4,193,071		
Region14	NRPS-like,betalactone	4,384,659	4,426,479	Thanamycin(NRP:Beta-lactam)	27%
Region 15	T1PKS,NRPS-like	4,660,340	4,707,922		
Region 16	opine-like-metallophore	5,789,089	5,811,178	Pseudopaline(Other)	100%

Table S9. The secondary metabolism gene clusters in the genome of *P. aeruginosa* MR48 predicted by AntiSMASH

Region	Type	From	To	Most similar known cluster(Type)	Similarity
Region 1	RiPP-like	917,925	932,794	pseudopyronine A/pseudopyronine (Other:Fatty acid)	12%
Region 2	redox-cofactor	1,802,454	1,824,616	lankacidin C(NRP+Polyketide)	13%
Region 3	NAGGN	2,647,999	2,662,791		
Region 4	ranthipeptide	2,741,160	2,762,590	Pf-5 pyoverdine(NRP)	4%
Region 5	NRPS	2,832,399	2,885,421	azotobactin D(NRP)	58%
Region 6	T3PKS,CDPS	3,063,489	3,122,628	2,4-diacetylphloroglucinol(Polyketid)	100%
Region 7	methanobactin	3,183,773	3,205,147	methanobactin(RiPP)	66%
Region 8	NRP-metallophore,NRPS	3,363,950	3,440,326	Pf-5 pyoverdine(NRP)	9%
Region 9	butyrolactone	4,594,247	4,607,603		
Region 10	arylpolyene	5,607,940	5,651,524	APE Vf (Other)	45%

Table S10. The same orthologous clusters identified in the *P. aeruginosa* SF416 and M18 genomes

cluster id	M18	SF416	Go annotation
cluster5606	WP_014603450.1	WP_003100208.1	GO:0016491; F:oxidoreductase activity; IEA:UniProtKB-KW
cluster5612	WP_003162920.1	WP_033937163.1	GO:0043571; P:maintenance of CRISPR repeat elements; IEA:InterPro
cluster5613	WP_016852510.1	WP_034037320.1	GO:0055085; P:transmembrane transport; IEA:InterPro
cluster5617	WP_014602883.1	WP_033937165.1	GO:0046872; F:metal ion binding; IEA:UniProtKB-KW
cluster5618	WP_003117661.1	WP_003160094.1	GO:0009089; P:lysine biosynthetic process via diaminopimelate; IEA:UniProtKB-UniPathway
cluster5633	WP_014602790.1	WP_003116715.1	GO:0006388; P:tRNA splicing, via endonucleolytic cleavage and ligation; IBA:GO_Central
cluster5635	WP_003104048.1	WP_003104048.1	GO:0019439; P:aromatic compound catabolic process; IDA:UniProtKB
cluster5646	WP_003162915.1	WP_003162915.1	GO:0043571; P:maintenance of CRISPR repeat elements; IEA:UniProtKB-UniRule
cluster5647	WP_014602746.1	WP_034037392.1	GO:0022857; F:transmembrane transporter activity; IEA:UniProtKB-UniRule
cluster5649	WP_014603449.1	WP_003100204.1	GO:0046244; P:salicylic acid catabolic process; IDA:UniProtKB
cluster5652	WP_003162432.1	WP_019681477.1	GO:0043565; F:sequence-specific DNA binding; IEA:InterPro
cluster5670	WP_003162431.1	WP_023083344.1	GO:0032440; F:2-alkenal reductase [NAD(P)] activity; IEA:UniProtKB-EC
cluster5672	WP_003100219.1	WP_003100219.1	GO:0009852; P:auxin catabolic process; IEA:EnsemblPlants

Table S11. The unique orthologous clusters in the *P. aeruginosa* SF416 genome

Cluster ID	Number of proteins	Proteins ID
cluster5468	3	WP_225025411.1
		WP_071535131.1
		WP_202965877.1
cluster5654	2	WP_061303886.1
		WP_083242027.1
cluster5655	2	WP_124034331.1
		WP_126632918.1
cluster5656	2	WP_161564154.1
		WP_012613973.1
